# The alerting expression of microRNA-411 predicts clinical prognosis and regulates tumor progression of glioblastoma

**DOI:** 10.1080/21655979.2021.1988365

**Published:** 2021-10-18

**Authors:** Weihua Fan, Xia Yu, Kunrong Li, Mingtao Zhu

**Affiliations:** a Department of Laboratory, Shandong Provincial Hospital Affiliated to Shandong First Medical University, Jinan, Shandong, China; b Department of Anesthesia and Perioperative Medicine, Dongying Hospital of Traditional Chinese Medicine, Dongying, Shandong, China; c Department of Neurosurgery, The First Affiliated Hospital of Xiamen University, Xiamen, Fujian, China

**Keywords:** miR-411, glioblastoma, tumor progression, prognosis

## Abstract

Glioblastoma is a malignant intracranial tumor with indispensable growth. Identification of biomarkers associated with the progression of tumors could benefit the clinical therapy of and improve patient’s survival. miR-411 has been reported to play a role in other cancers, while its function in glioblastoma has been explored in the present study. The expression of miR-411 was evaluated in glioblastoma tissues (collected from 108 glioblastoma patients) and cells by polymerase chain reaction. The clinical significance of miR-411 was estimated with a series of statistical analyses. The biological function of miR-411 in the cellular processes of glioblastoma was assessed by cell counting kit 8 and Transwell assay. The expression of miR-411 was significantly reduced in glioblastoma, which was associated with the Karnofsky Performance Score (KPS) and Isocitrate dehydrogenase 1 (IDH1) status of patients. miR-411 was identified as an independent prognostic indicator that correlated with the poor prognosis of patients. miR-411 suppressed the growth, migration, and invasion of glioblastoma cells via modulating signal transducer and activator of transcription 3 (STAT3). miR-411 participated in the development of glioblastoma and function as a prognostic biomarker. miR-411 functions as a tumor suppressor, which provides a novel potential therapeutic target for glioblastoma.

## Introduction

Glioblastoma is a kind of aggressive and lethal brain tumor in humans [1,2]. Surgical resection, adjuvant radiotherapy, and chemotherapy are the major treatments of glioblastoma [3]. Although the therapeutic strategy of glioblastoma has been improved remarkably, glioblastoma is still incurable because of the resistance to conventional treatments and postoperative metastasis and relapse [4,5]. The survival of patients is therefore unsatisfactory. Tumor progression is a complicated process involving various mechanisms and molecules. Recently, the identification of functional molecules that participate in tumor progression has attracted special attention, which could help improve the treatment efficiency and clinical prognosis.

The functions of microRNAs (miRNAs) have been widely reported in various human cancers, especially miRNAs with abnormal expression. miRNAs are short non-coding RNAs with a length of 18–25 nt. Previously, miRNAs have been evidenced to regulate the growth, differentiation, migration, and invasion of various cells. These cellular processes are also crucial factors for the development of tumors. In glioblastoma, several miRNAs were identified as potential biomarkers for disease onset and progression, such as miR-589-3p, miR-758-5p, and miR-185 [4,6,7]. miR-411 was also revealed to be a functional miRNA with altered expression in various cancers. For example, in gastric cancer and bladder cancer, miR-411 was illustrated to inhibit the disease development, while the promoted effect was found in the differentiation of osteoblast [8–10]. The dysregulation of miR-411 was also found in glioblastoma, but its function and clinical significance remain unknown [11].

The abnormal expression of miR-411 in glioblastoma implies its potential of acting as a biomarker in glioblastoma. The expression level of miRNAs was different in dissimilar cancers. The downregulation of miR-411 has been observed in renal cell carcinoma and breast cancer [12,13], and the upregulation was observed in lung cancer [14,15]. Therefore, the expression of miR-411 in glioblastoma needs to be validated. The aim of this study is to disclose the clinical significance and biological function of miR-411 in the development of glioblastoma.

## Materials and methods

### Patients and samples

The patient enrollment was conducted from 2012 to 2014, and this study had obtained approval from the Ethics Committee of the First Affiliated Hospital of Xiamen University (Approval NO. 20,111,222-17, 22 December 2011). One hundred and eight patients diagnosed with glioblastoma and underwent surgery were included in this study. The participants had never received any chemotherapy or radiotherapy before surgical treatment and signed informed consent. All patients were followed up for 5 years to summarize their survival information. Paired glioblastoma tissues and normal tissues were collected during surgery and frozen in liquid nitrogen for the following analyses.

### Cell incubation and cell transfection

U343, T98, A172, and LN229 cells were used as the typical glioblastoma cell lines, and the NHA cells were selected as the normal consultation cell lines. All cells were purchased from ATCC and maintained in the DMEM culture medium with 10% FBS (Sigma Aldrich). Cell culture was performed in a cell culture flask at 37°C with 5% CO_2_ [16].

Cells were transfected with miR-411 mimic, miR-411 inhibitor, or negative controls when they arrived the logarithmic growth phase with the employment of Lipofectamine 2000 (Invitrogen) according to the manufacturer’s instructions [17]. Transfected cells were available for the following experiments after 48 h of cell transfection.

### RT-qPCR

Collected tissues and treated cells were lysed to extract total RNA with TRIzol reagent. cDNA was generated from RNA with the help of reverse transcriptase (TaqMan MicroRNA RT Kit, Applied Biosystems). Then, cDNA was used for the quantification of miR-411 with the SYBR Green Master kit (Qiagen). The relative expression of miR-411 (primer sequences: forward 5ʹ-CCATGUAUGUAACACGGUCCAC-3ʹ and reverse: 5ʹ-GGUUAGUGGACCGGTCACC-3ʹ) was calculated with the 2^−ΔΔct^ method with U6 as the reference gene [18,19].

### Dual-luciferase reporter experiment

The wild-type and mutant-type STAT3 vectors were established by cloning binding sites or mutant sites into the pmirGLO plasmid. The co-transfection of established vectors and miR-411 mimic, miR-411 inhibitor, or negative controls was conducted with the Lipofectamine 2000 (Invitrogen) according to the manufacturer’s instructions. The luciferase activity of STAT3 was detected with the Dual-luciferase Reporter Assay System with the normalization to Renilla [20].

### In vitro cell experiments

Treated cells were seeded into 96-well plates and supplied with a complete culture medium. The seeded plates were incubated in an incubator at 37°C for a certain period of time. CCK8 reagent was added to each well, and the incubation was continued for 2 h. The absorbance at 450 nm was detected by a microplate reader to evaluate cell growth [17].

For the assessment of cell migration and invasion, treated cells were seeded into the upper chamber of 24-well transwell plates (BD Bioscience). The upper chamber was precoated with Matrigel (Corning) before the invasion assay. The seeded cells were supplied with a serum-free medium, and the bottom chamber was filled with a complete medium with FBS as the chemoattractant. The migration and invasion assay was conducted at 37°C for 24 h, then the migrated and invaded cells were stained with crystal violet and analyzed with an optical microscope (Olympus) [17].

### Statistical analysis

All data were obtained from independent triplicate experiments and expressed as a mean value ± SD. Data were analyzed using the SPSS 20.0 software. The difference between the two groups was estimated by Student’s t-test, and one-way ANOVA was used to evaluate the differences among multiple groups. The clinical significance of miR-411 was assessed with the Chi-square test, Kaplan–Meier analysis, and Cox regression analysis. The results were considered to be statistically significant when *P* < 0.05.

## Results

miR-411 was speculated to play a role in the progression of glioblastoma. The expression of miR-411 was evaluated in glioblastoma tissues and cells. The specific role of miR-411 in glioblastoma was estimated with a series of statistical analyses and *in vitro* cell experiments.

### The downregulation of miR-411 in glioblastoma tissues and cells

**Figure 1.**
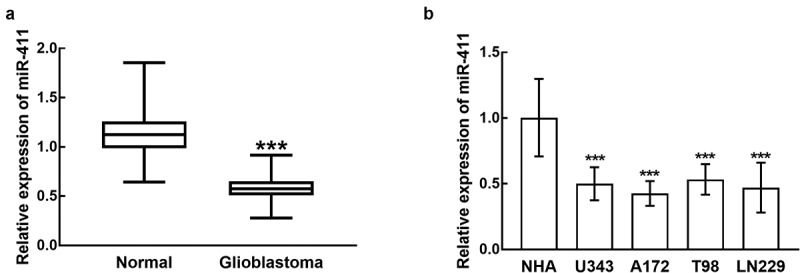
miR-411 was downregulated in glioblastoma tissues (a) and cells (b) compared with normal tissues and normal cells. ***P < 0.001

In glioblastoma tissues, miR-411 was dramatically reduced in comparison with normal tissues (*P* < 0.001, Figure 1a). Furthermore, in glioblastoma cells, the expression levels of miR-411 were much lower than that in normal cells, and the difference was significant (*P* < 0.001, Figure 1b). Moreover, due to the relatively high sensitivity of A172 and LN229 cells to miR-411 downregulation, these two cells were applied in the following cell experiments.

### The clinical significance of miR-411 in glioblastoma

Recruited patients were divided into two groups based on the mean value of the miR-411 expression in glioblastoma tissues. Patients in the low miR-411 expression group showed a lower Karnofsky Performance Score (KPS) and a wild type of IDH1. The significant association of miR-411 expression with the KPS (*P* = 0.018) and IDH1 (*P* = 0.019) status was demonstrated with the help of the Chi-square test (Table 1).

**Table 1.** Clinicopathological features associated with miR-411 expression in glioblastoma patients

	Total number (108)	miR-411 expression		*P* Value
		Low (55)	High (53)	
Age (years)				0.568
≤ 50	52	25	27	
>50	56	30	26	
Gender				0.583
Male	66	35	31	
Female	42	21	22	
KPS				0.018
≤80	71	42	29	
>80	37	13	24	
IDH1				0.019
Mutation	39	14	25	
Wild type	69	41	28	
Surgery				0.443
Total resection	53	25	28	
Partial resection	55	30	25	
Tumor size (cm)				0.181
≤5	50	22	28	
>5	58	33	28	
Family history				0.450
Negative	49	23	26	
Positive	59	32	27	

The IDH1 mutation was analyzed by PCR; The KPS (Karnofsky Performance Score) was evaluated according to the widely accepted criteria.

**Figure 2.**
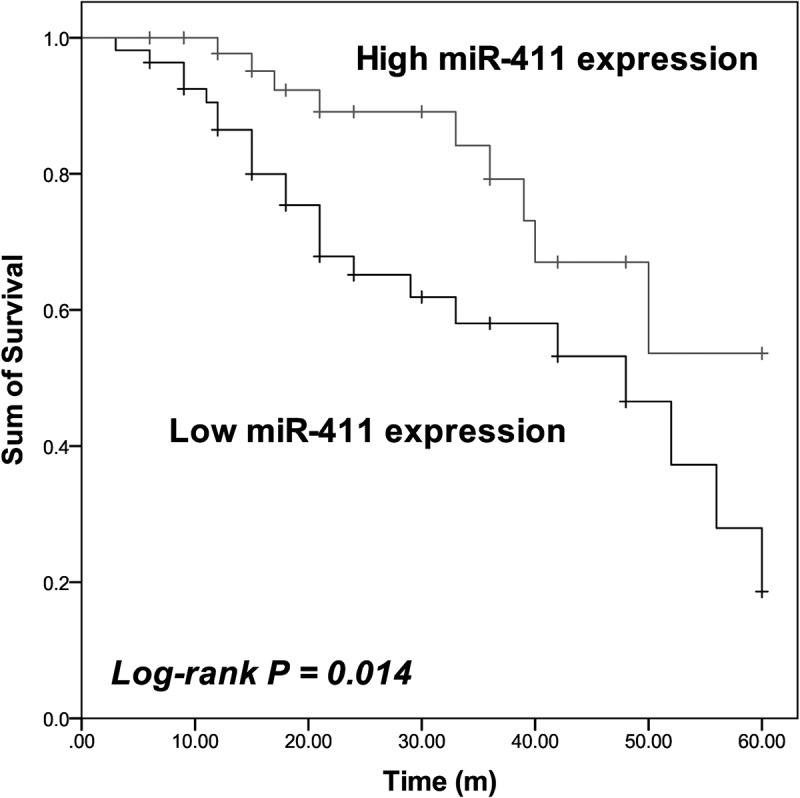
Patients with low miR-411 expression showed shorter survival than patients with high miR-411 expression. Log-rank P = 0.014

Additionally, the results of Kaplan–Meier analysis showed that patients in the low miR-411 expression group possessed a shorter lifetime than patients in the high miR-411 expression group (Log-rank *P* = 0.014, Figure 2). The Cox regression analysis also identified miR-411 as an independent prognostic biomarker with the HR value of 3.910 (95% CI = 1.598–9.564, *P* = 0.003) together with KPS (HR value = 3.078, 95% CI = 1.050–9.027, *P* = 0.041) and IDH1 status (HR value = 3.021, 95% CI = 1.324–6.892, *P* = 0.009) of patients (Table 2).

**Table 2.** Prognostic factors for the overall survival of glioblastoma patients by multivariate Cox regression analysis

	95% CI	HR Value	*P* value
miR-411	1.598–9.564	3.910	0.003
Age	0.63–2.860	1.349	0.436
Gender	0.473–2.411	1.068	0.874
KPS	1.050–9.027	3.078	0.041
IDH1	1.324–6.892	3.021	0.009
Surgery	0.581–2.550	1.214	0.603
Tumor size	0.773–3.593	1.667	0.192
Family history	0.761–3.741	1.688	0.198

KPS: Karnofsky Performance Score.

### The biological function of miR-411 in glioblastoma cells

**Figure 3.**
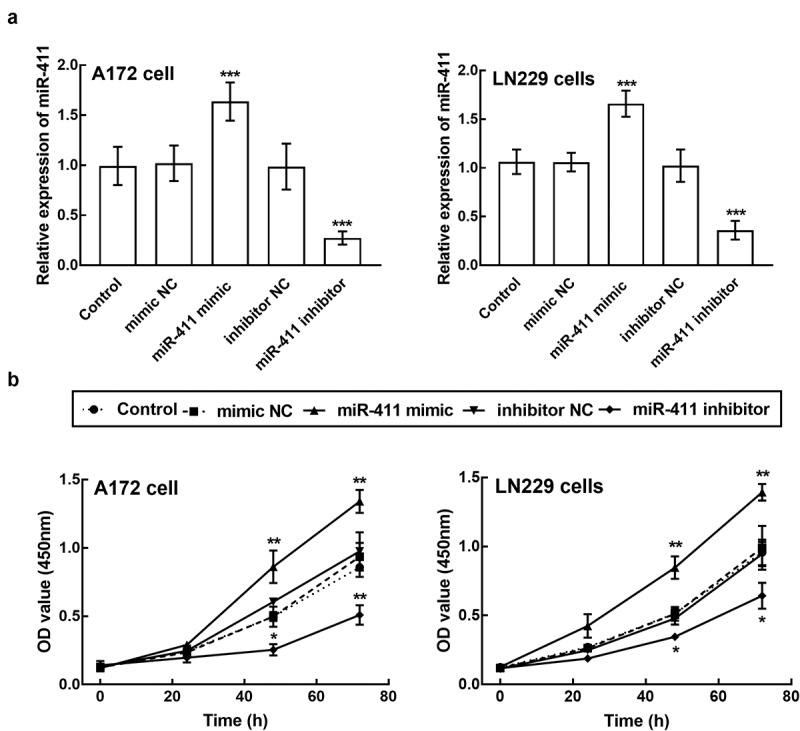
Effect of miR-411 on cell proliferation. **a**. The expression of miR-411 in A172 and LN229 cells was suppressed by the transfection of miR-411 inhibitor and enhanced by the transfection of miR-411 mimic. **b**. The overexpression of miR-411 significantly inhibited the proliferation of A172 and LN229 cells, which was promoted by the knockdown of miR-411. *P < 0.05, **P < 0.01, ***P < 0.001

A172 and LN229 cells were transfected with miR-411 mimic, which significantly enhanced the expression level of miR-411 (*P* < 0.001, Figure 3a). Meanwhile, these two cells were also transfected with miR-411 inhibitor, which suppressed the expression of miR-411 (*P* < 0.001, Figure 3a). The proliferation capacity of A172 and LN229 cells with miR-411 overexpression was dramatically poorer than that of untransfected cells and negative controls, whereas cells with miR-411 knockdown showed a much stronger proliferation ability (*P* < 0.05, *P* < 0.01, Figure 3b).

**Figure 4.**
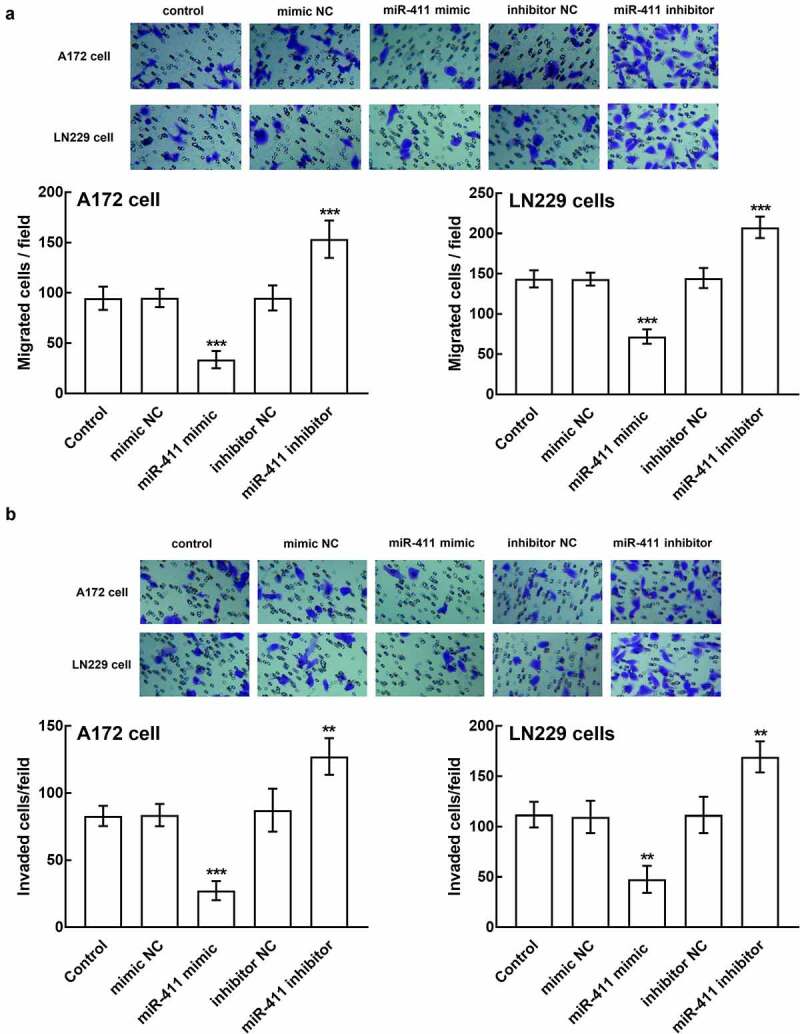
Effect of miR-411 on cell migration and invasion. **a**. The migration of A172 and LN229 cells was boosted by the downregulation of miR-411 and suppressed by the upregulation of miR-411. **b**. The invasion of A172 and LN229 cells was facilitated by miR-411 overexpression and inhibited by miR-411 knockdown. **P < 0.01, ***P < 0.001

Moreover, the regulatory effect of miR-411 was also observed in the migration and invasion of glioblastoma cells. In the presence of miR-411 overexpression, the number of migrated cells plummeted, which surged in the presence of miR-411 silencing (*P* < 0.001, Figure 4a). Similarly, the invasion of A172 and LN229 cells was also inhibited by the miR-411 upregulation and enhanced by the downregulation of miR-411, which is significant relative to the untransfected cells and negative controls (*P* < 0.01, *P* < 0.001, Figure 4b).

### The potential mechanism underlying the function of miR-411

**Figure 5.**
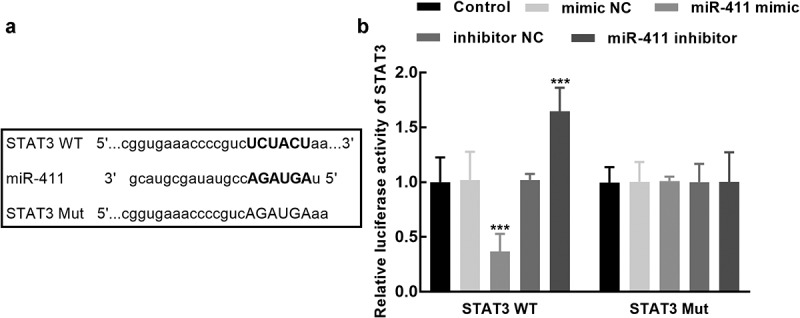
Assessment of the interaction between miR-411 and STAT3. **a**. The binding sites between miR-411 and STAT3 were predicted and cloned into the pmirGLO plasmid. **b**. The luciferase activity of STAT3 was significantly suppressed by the overexpression of miR-411 and enhanced by its knockdown. ***P < 0.001

In the STAT3 wild-type vector that contains the binding sites between miR-411 and STAT3 (Figure 5a), the luciferase activity was significantly suppressed by the overexpression of miR-411, which was dramatically enhanced by its silencing (*P* < 0.001, Figure 5b), whereas the luciferase activity of the STAT3 mutant-type vector was not affected by the expression of miR-411 (*P* > 0.05, Figure 5b).

## Discussion

Identification of novel molecules participating in the progression of glioblastoma and possessing the ability to regulate cell processes is of great interest in cancer research. In previous studies, the function of miRNAs has been widely investigated. For example, miR-1238 was reported to play a vital role in the treatment of glioblastoma as the fact that miR-1238 mediated the chemoresistance of glioblastoma and CAV1/EGFR was revealed to be the direct target during its functioning [21]. miR-449-5p was disclosed as a downregulated miRNA in glioblastoma and was also identified as a tumor suppressor that retarded the occurrence of glioblastoma [22]. Herein, the role of miR-411 in glioblastoma is studied.

The dysregulation of miR-411 was previously found in the expression profile of differentially expressed miRNAs in glioblastoma compared with non-tumor brain tissues by Skalsky et al. [11]. Due to the fact that the expression of miRNAs varies from sample to sample, the expression levels of miR-411 were validated in glioblastoma tissues and cells, and a significant downregulation was observed. The downregulation of miR-411 was correlated with the low KPS and wild type of IDH1 of patients. KPS is an important factor in the assessment of patient’s clinical status and is associated with the development of glioblastoma [23]. KPS is also routinely used as a prognostic indicator to estimate the functional independence of tumor patients perioperatively [24]. IDH1 was also identified as a favorable prognostic factor for glioblastoma. The clinical significance of IDH1 was first illustrated by Yan et al., that the mutation of IDH1 indicated a better outcome [25]. The close association between miR-411 and KPS and IDH1 of patients in the present study implies the potential involvement of miR-411 in the progression and prognosis of glioblastoma. Additionally, consistent with previous studies, miR-411, as well as KPS and IDH1, was revealed to function as an independent prognostic indicator for glioblastoma.

miR-411 was also indicated to be involved in the development of many other cancers and regulate various major cellular processes. For instance, the downregulation of miR-411 was found in renal cell carcinoma, which showed a significantly inhibitory effect on cell growth and migration and induced effect on cell apoptosis [12]. Otherwise, a significant upregulation of miR-411 was found in hepatocellular carcinoma, which promoted the proliferation and anchorage-independent growth of hepatocellular carcinoma cells via suppressing ITCH, a putative tumor suppressor [26,27]. miR-411 was reported to mediate the regulatory effect of lncRNA PSMA3-AS1 and lncRNA ECONEXIN in glioma [28,29]. However, studies focused on the specific function of miR-411 lacked. In the present study, the downregulation of miR-411 was found to promote cell proliferation, migration, and invasion of glioblastoma, while its overexpression showed opposite effects. These results indicated the tumor suppressor role of miR-411 in glioblastoma.

Moreover, the mechanism underlying the function of miR-411 could benefit the clinical application of miR-411 prognostic biomarker and tumor suppressor function. Numerous molecules have been confirmed as targets of miR-411 during its biological effect on various tumor progressions. Previously, the interaction between miR-411 and STAT3 has been demonstrated to be the mechanism underlying the tumor suppressor role of miR-411 in cervical cancer [30]. STAT3 was also identified as the direct target of many other tumor suppressors of glioblastoma, and STAT3 has also been indicated to be upregulated in glioblastoma [31–33]. Here, the interaction between miR-411 and STAT3 was validated with a dual-luciferase reporter assay. miR-411 could modulate the luciferase activity of STAT3, which was speculated to be the molecular mechanism during miR-411 regulating glioblastoma progression.Though the present study provides direct evidence to verify the functional role of miR-411 in glioblastoma, there are still some minor limitations. The relatively small sample size limited the clinical results to some degree. Among clinicopathological features of patients, except for KPS and IDH1, other characteristics are also crucial factors associated with the development of glioblastoma. However, no significant relationship was found between miR-411 and those features, which might be a result of the limited sample size.

## Conclusion

The downregulation of miR-411 in glioblastoma was correlated with the disease development and clinical outcome of patients. miR-411 serves as a tumor suppressor, which might be a novel therapeutic target of glioblastoma.

## References

[1] Dolecek TA, Propp JM, Stroup NE, et al. CBTRUS statistical report: primary brain and central nervous system tumors diagnosed in the United States in 2005-2009. Neuro Oncol. 2012;14(Suppl 5):v1–49. 23095881 10.1093/neuonc/nos218 PMC3480240

[2] Kotliarova S, Fine HA. SnapShot: glioblastoma multiforme. Cancer Cell. 2012;21(5):710–710 e1. 22624719 10.1016/j.ccr.2012.04.031

[3] Khosla D. Concurrent therapy to enhance radiotherapeutic outcomes in glioblastoma. Ann Transl Med. 2016;4(3):54. 26904576 10.3978/j.issn.2305-5839.2016.01.25 PMC4740000

[4] Cesarini V, Silvestris DA, Tassinari V, et al. ADAR2/miR-589-3p axis controls glioblastoma cell migration/invasion. Nucleic Acids Res. 2018;46(4):2045–2059. 29267965 10.1093/nar/gkx1257 PMC5829642

[5] Lefranc F, Sadeghi N, Camby I, et al. Present and potential future issues in glioblastoma treatment. Expert Rev Anticancer Ther. 2006 May;6(5):719–732. 16759163 10.1586/14737140.6.5.719

[6] Liu A, Shen Y, Du Y, et al. Esculin prevents Lipopolysaccharide/D-Galactosamine-induced acute liver injury in mice. Microb Pathog. 2018 Dec;125:418–422. 30290266 10.1016/j.micpath.2018.10.003

[7] Wu W, Yu T, Wu Y, et al. The miR155HG/miR-185/ANXA2 loop contributes to glioblastoma growth and progression. J Exp Clin Cancer Res. 2019 Mar 21;38(1):133. 30898167 10.1186/s13046-019-1132-0 PMC6427903

[8] Bai TL, Liu YB, Li BH. MiR-411 inhibits gastric cancer proliferation and migration through targeting SETD6. Eur Rev Med Pharmacol Sci. 2019 Apr;23(8):3344–3350. 31081088 10.26355/eurrev_201904_17697

[9] Gao X, Ge J, Li W, et al. Over-expression of miR-411-5p and miR-434-3p promotes the osteoblast differentiation by targeting GATA4. Mol Cell Endocrinol. 2020;506:110759. 32061766 10.1016/j.mce.2020.110759

[10] Liu Y, Liu T, Jin H, et al. MiR-411 suppresses the development of bladder cancer by regulating ZnT1. Onco Targets Ther. 2018;11:8695–8704. 30584327 10.2147/OTT.S173750 PMC6287661

[11] Skalsky RL, Cullen BR. Reduced expression of brain-enriched microRNAs in glioblastomas permits targeted regulation of a cell death gene. PLoS One. 2011;6(9):e24248. 21912681 10.1371/journal.pone.0024248 PMC3166303

[12] Zhang X, Zhang M, Cheng J, et al. MiR-411 functions as a tumor suppressor in renal cell cancer. Int J Biol Markers. 2017 Oct 31;32(4):e454–e460. 28708205 10.5301/ijbm.5000261

[13] Zhang Y, Xu G, Liu G, et al. miR-411-5p inhibits proliferation and metastasis of breast cancer cell via targeting GRB2. Biochem Biophys Res Commun. 2016 Aug 5;476(4):607–613. 27264952 10.1016/j.bbrc.2016.06.006

[14] Wang SY, Li Y, Jiang YS, et al. Investigation of serum miR-411 as a diagnosis and prognosis biomarker for non-small cell lung cancer. Eur Rev Med Pharmacol Sci. 2017 Sep;21(18):4092–4097. 29028091

[15] Zhang C, Wang H, Liu X, et al. Oncogenic microRNA-411 promotes lung carcinogenesis by directly targeting suppressor genes SPRY4 and TXNIP. Oncogene. 2019 Mar;38(11):1892–1904. 30390072 10.1038/s41388-018-0534-3 PMC6475890

[16] Bhargava S, Patil V, Mahalingam K, et al. Elucidation of the genetic and epigenetic landscape alterations in RNA binding proteins in glioblastoma. Oncotarget. 2017 Mar 7;8(10):16650–16668. 28035070 10.18632/oncotarget.14287 PMC5369992

[17] Guo L, Yuan J, Xie N, et al. miRNA-411 acts as a potential tumor suppressor miRNA via the downregulation of specificity protein 1 in breast cancer. Mol Med Rep. 2016 Oct;14(4):2975–2982. 27572271 10.3892/mmr.2016.5645 PMC5042781

[18] Livak KJ, Schmittgen TD. Analysis of relative gene expression data using real-time quantitative PCR and the 2(-Delta Delta C(T)) Method. Methods. 2001;25(4):402–408. 11846609 10.1006/meth.2001.1262

[19] Rao X, Huang X, Zhou Z, et al. An improvement of the 2^(-delta delta CT) method for quantitative real-time polymerase chain reaction data analysis. Biostat Bioinforma Biomath. 2013 Aug;3(3):71–85. 25558171 PMC4280562

[20] Clement T, Salone V, Rederstorff M. Dual luciferase gene reporter assays to study miRNA function. Methods Mol Biol. 2015;1296:187–198. 25791601 10.1007/978-1-4939-2547-6_17

[21] Yin J, Zeng A, Zhang Z, et al. Exosomal transfer of miR-1238 contributes to temozolomide-resistance in glioblastoma. EBioMedicine. 2019 Apr;42:238–251. 30917935 10.1016/j.ebiom.2019.03.016 PMC6491393

[22] Hou WZ, Chen XL, Qin LS, et al. MiR-449b-5p inhibits human glioblastoma cell proliferation by inactivating WNT2B/Wnt/beta-catenin signaling pathway. Eur Rev Med Pharmacol Sci. 2020 May;24(10):5549–5557. 32495889 10.26355/eurrev_202005_21340

[23] Stark AM, Stepper W, Mehdorn HM. Outcome evaluation in glioblastoma patients using different ranking scores: KPS, GOS, mRS and MRC. Eur J Cancer Care (Engl). 2010 Jan 1;19(1):39–44. 19912295 10.1111/j.1365-2354.2008.00956.x

[24] Sacko A, Hou MM, Temgoua M, et al. Evolution of the Karnofsky Performance Status throughout life in glioblastoma patients. J Neurooncol. 2015 May;122(3):567–573. 25700836 10.1007/s11060-015-1749-6

[25] Yan H, Parsons DW, Jin G, et al. IDH1 and IDH2 mutations in gliomas. N Engl J Med. 2009 Feb 19;360(8):765–773. 19228619 10.1056/NEJMoa0808710 PMC2820383

[26] Xia K, Zhang Y, Cao S, et al. miR-411 regulated ITCH expression and promoted cell proliferation in human hepatocellular carcinoma cells. Biomed Pharmacother. 2015 Mar;70:158–163. 25776495 10.1016/j.biopha.2015.01.001

[27] Li Y, Ge YZ, Xu L, et al. Circular RNA ITCH: a novel tumor suppressor in multiple cancers. Life Sci. 2020;254:117176. 31843532 10.1016/j.lfs.2019.117176

[28] Huang T, Chen Y, Zeng Y, et al. Long non-coding RNA PSMA3-AS1 promotes glioma progression through modulating the miR-411-3p/HOXA10 pathway. BMC Cancer. 2021 Jul 22;21(1):844. 34294084 10.1186/s12885-021-08465-5 PMC8296684

[29] Deguchi S, Katsushima K, Hatanaka A, et al. Oncogenic effects of evolutionarily conserved noncoding RNA ECONEXIN on gliomagenesis. Oncogene. 2017 Aug 10;36(32):4629–4640. 28368417 10.1038/onc.2017.88

[30] Shan D, Shang Y, Hu T. MicroRNA-411 inhibits cervical cancer progression by directly targeting STAT3. Oncol Res. 2019 Feb 21;27(3):349–358. 29716674 10.3727/096504018X15247361080118 PMC7848402

[31] Han D, Yu T, Dong N, et al. Napabucasin, a novel STAT3 inhibitor suppresses proliferation, invasion and stemness of glioblastoma cells. J Exp Clin Cancer Res. 2019 Jul 5;38(1):289. 31277685 10.1186/s13046-019-1289-6 PMC6612138

[32] Kim BH, Lee H, Park CG, et al. STAT3 inhibitor ODZ10117 suppresses glioblastoma malignancy and prolongs survival in a glioblastoma xenograft model. Cells. 2020 15;9(3):Mar. 10.3390/cells9030722 PMC7140655 32183406

[33] Piperi C, Papavassiliou KA, Papavassiliou AG. Pivotal role of STAT3 in shaping glioblastoma immune microenvironment. Cells. 2019 6;8(11):Nov. 10.3390/cells8111398 PMC6912524 31698775

